# How the Pathological Microenvironment Affects the Behavior of Mesenchymal Stem Cells in the Idiopathic Pulmonary Fibrosis

**DOI:** 10.3390/ijms21218140

**Published:** 2020-10-30

**Authors:** Martina Bonifazi, Mariangela Di Vincenzo, Miriam Caffarini, Federico Mei, Michele Salati, Lina Zuccatosta, Majed Refai, Monica Mattioli-Belmonte, Stefano Gasparini, Monia Orciani

**Affiliations:** 1 Department of Biomedical Sciences and Public Health, Universitá Politecnica delle Marche, 60126 Ancona, Italy; m.bonifazi@univpm.it (M.B.); Federico.Mei@ospedaliriuniti.marche.it (F.M.); lina.zuccatosta@ospedaliriuniti.marche.it (L.Z.); s.gasparini@univpm.it (S.G.); 2 Pulmonary Diseases Unit, Department of Internal Medicine, Azienda Ospedaliero-Universitaria “Ospedali Riuniti”, 60126 Ancona, Italy; 3 Department of Clinical and Molecular Sciences—Histology, Università Politecnica delle Marche, 60126 Ancona, Italy; m.divincenzo@univpm.it (M.D.V.); m.caffarini@pm.univpm.it (M.C.); m.mattioli@univpm.it (M.M.-B.); 4 Unit of Thoracic Surgery, AOU Ospedali Riuniti, 60126 Ancona, Italy; michele.salati@ospedaliriuniti.marche.it (M.S.); majed.refai@ospedaliriuniti.marche.it (M.R.)

**Keywords:** mesenchymal stem cells, idiopathic pulmonary fibrosis, paracrine effect, inflammation

## Abstract

Idiopathic pulmonary fibrosis (IPF) is a chronic disease characterized by fibroblasts activation, ECM accumulation, and diffused alveolar inflammation. The role of inflammation in IPF is still controversial and its involvement may follow nontraditional mechanisms. It is seen that a pathological microenvironment may affect cells, in particular mesenchymal stem cells (MSCs) that may be able to sustain the inflamed microenvironment and influence the surrounding cells. Here MSCs have been isolated from fibrotic (IPF-MSCs) and control (C-MSCs) lung tissue; first cells were characterized and compared by the expression of molecules related to ECM, inflammation, and other interdependent pathways such as hypoxia and oxidative stress. Subsequently, MSCs were co-cultured between them and with NHLF to test the effects of the cellular crosstalk. Results showed that pathological microenvironment modified the features of MSCs: IPF-MSCs, compared to C-MSCs, express higher level of molecules related to ECM, inflammation, oxidative stress, and hypoxia; notably, when co-cultured with C-MSCs and NHLF, IPF-MSCs are able to induce a pathological phenotype on the surrounding cell types. In conclusion, in IPF the pathological microenvironment affects MSCs that in turn can modulate the behavior of other cell types favoring the progression of IPF.

## 1. Introduction

Idiopathic pulmonary fibrosis (IPF) is a chronic, progressive, and fatal interstitial lung disease (ILD) [1], characterized by an increase of fibroblast proliferation, an excessive extracellular matrix (ECM) deposition [2], and distortion of lung architecture, eventually leading to respiratory failure [3]. To date, limited treatment options are available, and prognosis remains still poor [4]. Although the etiology of this disease is not completely known, some studies have shown how environmental factors (smoking and exposure to toxic agents), age, and genetic factors represent the main risk factors for IPF onset [5]. In recent years several studies have agreed that IPF does not appear to be the direct result of immune cell dysfunction but rather that immune and inflammatory cells may play a role in fibroproliferation [6]. In fact, observations from clinical studies and experimental models have shown that the stimuli affecting the pulmonary epithelial cells (AEC type II) lead to the activation of fibroblasts in myofibroblast and the accumulation of ECM that can occur without a primary immunopathogenic component [7]. Once the fibrotic response is established, resident and recruited immune cells, such as macrophages and lymphocytes, modulate existing responses through a variety of mechanisms. Even if many clinical observations [8] suggest the dominant role of fibroblast dysfunction in IPF as opposed to an inflammatory process, inflammation remains a critical factor in IPF through non-traditional mechanisms.

Up to five different hypotheses for inflammation involvement in IPF have been proposed [8] (direct inflammatory hypothesis, matrix hypothesis, growth factor–receptor hypothesis, plasticity hypothesis, and vascular hypothesis) and all of them share the role of the paracrine effect exerted by the secretion of cytokines. In addition, the link between inflammation and oxidative stress as well as with hypoxia is very complex: inflammation has an interdependent relationship with hypoxia and oxidative stress [9,10] that have been already identified as inducers of fibrosis in IPF [11,12].

It has been well documented that mesenchymal stem cells (MSCs) secrete a wide pattern of pro- and anti-inflammatory cytokines [13]; in addition, previous works [14,15] underlined as the molecular markers found in the differentiated cells of some pathologies were already present at the staminal level, backdating the onset of the pathology itself. MSCs have been isolated from lung tissue of patients with IPF, but little is actually known about their role on IPF onset and/or development [16,17]. MSCs isolated from bone marrow have been also employed as potential treatment for lung injury and fibrosis showing that MSCs can migrate to injured sites, regulate endothelial and epithelial permeability, reduce inflammation, and improve tissue repair [18,19]. Here, MSCs were isolated from lung of patients affected by IPF (IPF-MSC), characterized according to the criteria by Dominici [20] and compared with MSCs isolated from healthy pulmonary tissue (C-MSC). Subsequently, IPF-MSCs were co-cultured with C-MSCs and with a fibroblast lung cell line (NHLF) and the expression of molecules differently involved in inflammation were analyzed before and after co-cultures to evaluate the effects of IPF-MSC on other cells. Therefore, the main aims of the present study are to evaluate how (i) the pathological microenvironment may affect MSCs behavior and (ii) dysregulated MSCs may in turn act on surrounding cells favoring the onset of this pathology.

## 2. Results

### 2.1. MSCs Isolation and Characterization from Healthy and Fibrotic Lung

**Figure 1:**
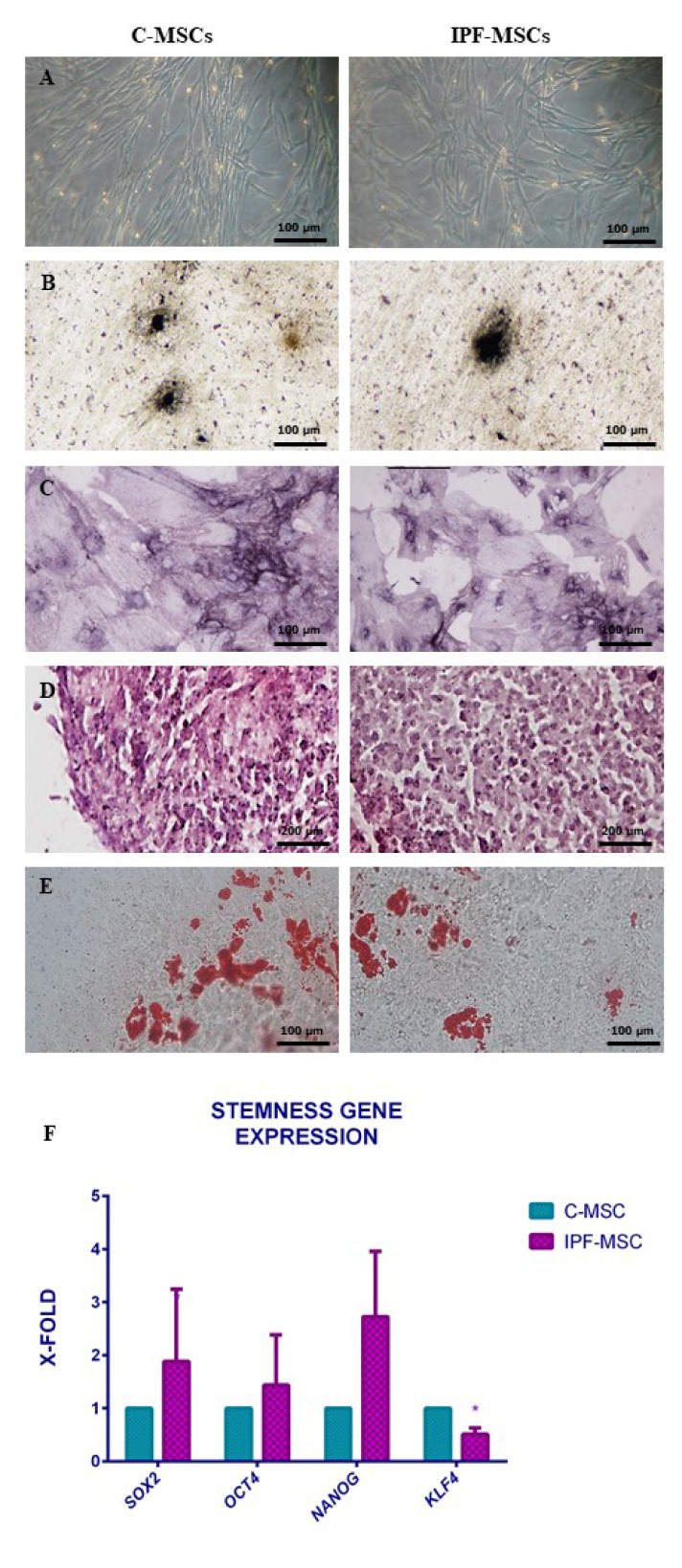
Mesenchymal stem cells (MSCs) characterization. Phase-contrast images (**A**) of MSCs derived from control lung tissue (C-MSCs) and from lung of patients affected by idiopathic pulmonary fibrosis (IPF) (IPF-MSCs) after 14 days of culture (scale bar, 100 μm); representative images of osteogenic differentiation assessment by von Kossa staining (**B**, scale bar 100 μm) and alkaline phosphatase (ALP) reaction (**C,** scale bar 100 μm); chondrogenic differentiation by safranin-O coloration (**D**, scale bar 200 μm); adipocyte differentiation by oil red staining (**E**, scale bar 30 μm). The histogram (**F**) depicts the expression of genes related to stemness. Levels of expression detected in IPF-MSCs are referred as X-fold with respect to C-MSCs (expressed equal to 1). Data are means ± SD from analyses performed on three separate experiments in triplicates. The * indicates significative differences between C-MSCs and IPF-MSC (unpaired *t*-test; *p* < 0.05).

Lung tissues from three patients affected by IPF and from three patients undergoing lobectomy for adenocarcinoma (intended as controls limited to the IPF pathology) were used to establish cell cultures. Cells derived from IPF samples were named IPF-MSCs while the others served as controls (C-MSCs). Cells appeared homogeneous, with a fibroblastoid morphology (Figure 1A). All subsequent experiments were performed separately on each cell sample. Since no differences were detected among the samples from the two tissue groups, no pair wise analysis was necessary and values are the average of samples. Evaluation of the stemness criteria identified by Dominici et al. [20] demonstrated that cells adhered to plastic and were also capable of differentiating toward osteogenic, chondrogenic, and adipogenic lineages. Osteogenic differentiation was detected after 10 days; cells appeared strongly positive for Von Kossa staining showing aggregates of mineralized matrix (Figure 1B) and alkaline phosphatase activity (ALP) (Figure 1C). Chondrogenic differentiation was achieved after 30 days, as shown by safranin-O staining (Figure 1D). MSCs differentiation into adipocytes occurred after 15 days, as evidenced by the accumulation of lipid vacuoles within the cytoplasm after oil red staining (Figure 1E). No significant differences were noted among the six cultures of MSCs. Stemness evaluation was integrated with the analysis of the expression of specific genes; no significative differences were founded between C and IPF-MSCs for *SOX2*, *OCT4*, *NANOG* gene expression, while the expression of *KLF4* was lower in IPF-MSCs than in C-MSCs (Figure 1F).

**Figure 2:**
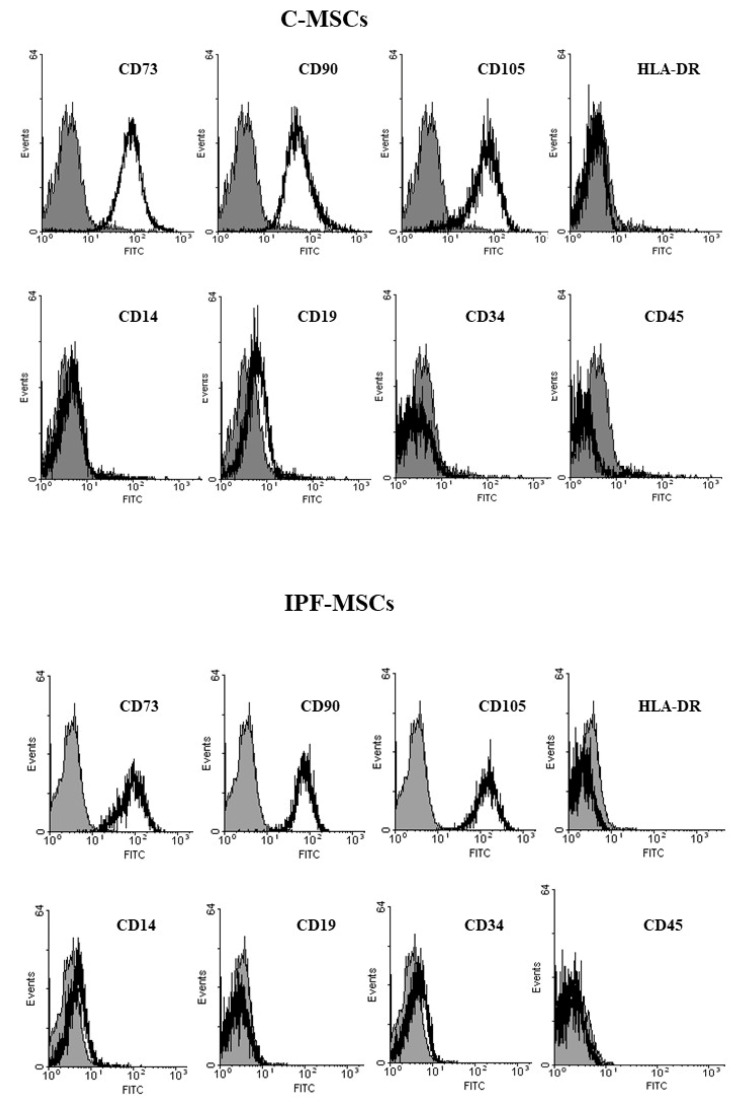
Immunophenotype of Mesenchymal stem Cells (MSCs). Representative FACScan analyses of cell-surface antigen expression, as indicated. Solid gray histograms refer to the negative control (IgG1 isotype control-FITC labeled). No differences were observed between MSCs isolated from the different subgroups.

Finally, cells were strongly positive for CD73, CD90, and CD105 and negative for HLA-DR, CD14, CD19, CD34, CD45, and CD9 (Figure 2).

### 2.2. α-SMA, Collagen Type 1, Fibronectin, and TGF-β1 Expression

**Figure 3:**
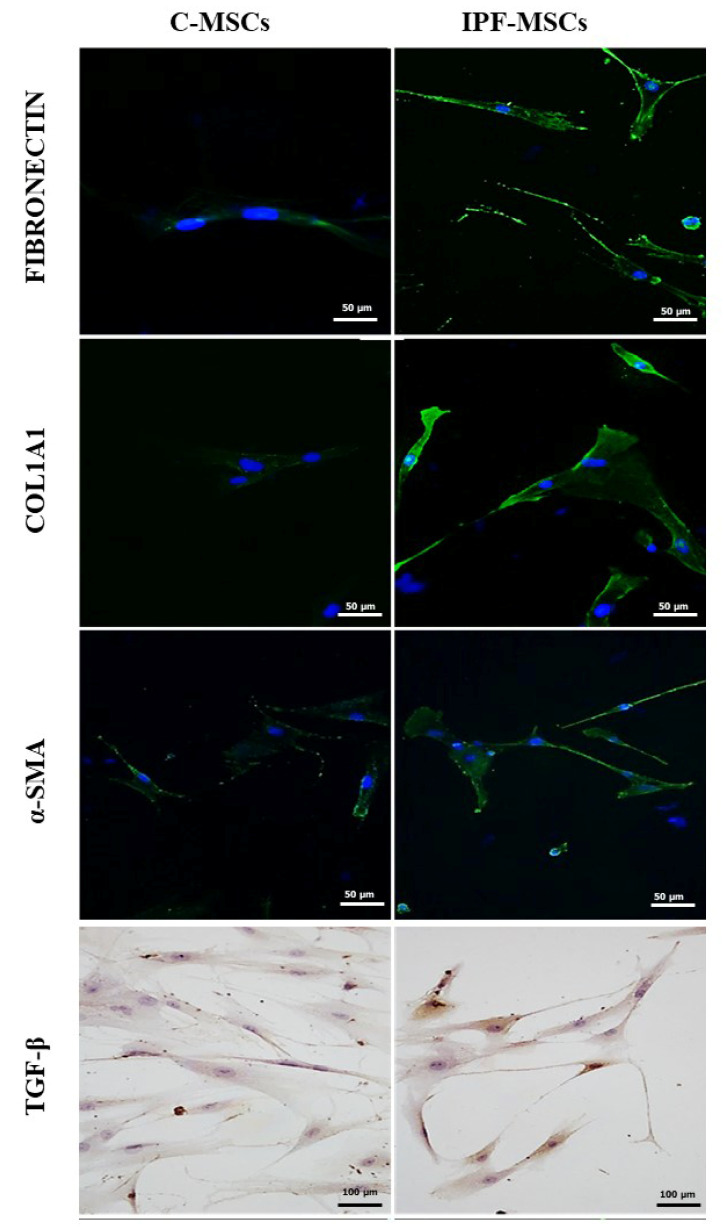
α-SMA, collagen type 1, fibronectin, and TGF-β1 expression. Indirect immunofluorescence (IIF) analysis of α-SMA, collagen type 1 (COL1A1), and fibronectin and immunocytochemical (ICC) analysis of TGF-β1 performed on mesenchymal stem cells (MSCs) derived from control (C-MSCs) and idiopathic pulmonary fibrosis (IPF-MSCs) tissues. For IIF, a secondary FITC-conjugated antibody was used after incubation with the primary antibodies. Nuclei were counterstained with Hoechst 33342. For ICC of TGF-β1, slides were treated with 3,3-diaminobenzidine and counterstained with Mayer’s hematoxylin.

Since it has been demonstrated that MSCs from pathological tissues acquire specific features [21], the expression of the main molecules characterizing IPF was analyzed in IPF-MSCs and C-MSCs. The expression of α-SMA, collagen type 1 (COL1A1), fibronectin, and TGF-β1 was faint in C-MSCs whereas IPF-MSCs showed a strong expression (Figure 3). The percentage of cells area occupied by the proteins, calculated using Fiji-ImageJ software (available online at https://imagej.net/Fiji) [22], in IPF-MSCs was significantly higher than C-MSC (Table 1).

### 2.3. Cell Proliferation: Differences between Mock and Co-Cultured Cells

**Figure 4:**
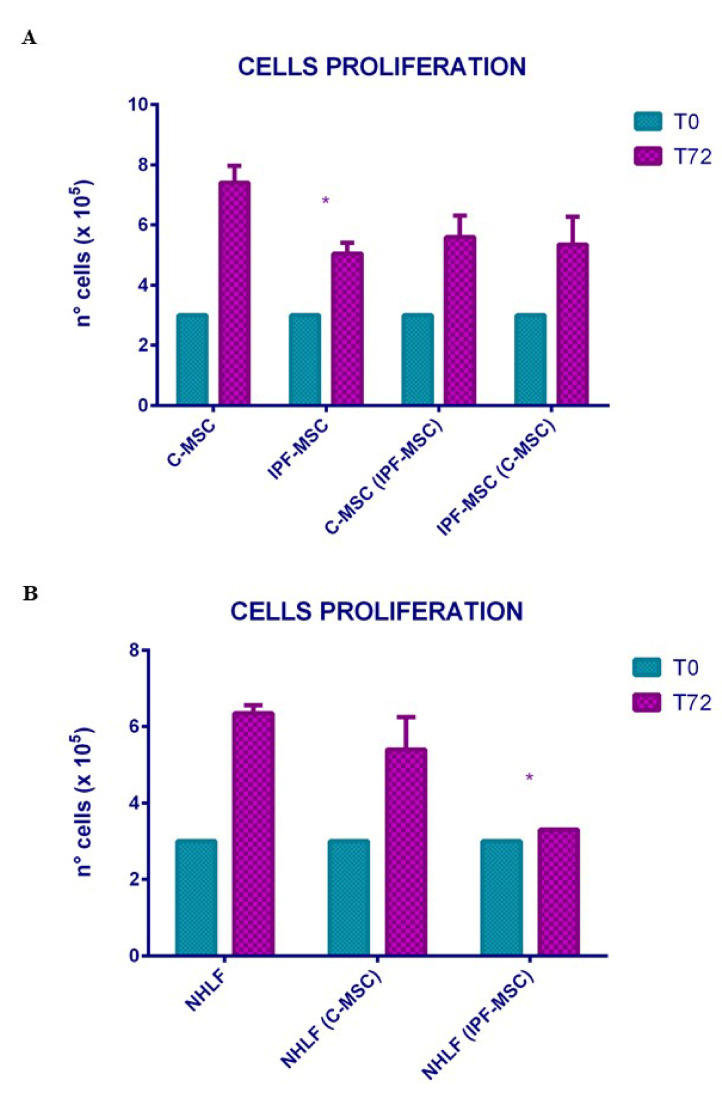
Effect of co-cultures on cellular proliferation. The histogram (**A**) indicates the proliferation of mesenchymal stem cells (MSCs) derived from control (C-MSCs) and idiopathic pulmonary fibrosis (IPF-MSCs) tissues (cultured alone) or co-cultured. C-MSCs: MSCs from controls cultured alone; IPF-MSCs: MSCs from patients with IPF cultured alone; C-MSC (IPF-MSCs): C-MSCs after 72 h of co-culture with IPF; IPF-MSCs (C-MSCs): IPF-MSCs after 72 h of co-culture with C-MSCs. The * indicate significative differences referred to C-MSC (one-way ANOVA; *p* < 0.05). Histogram (**B**) depicts the proliferation of NHLF cultured alone or co-cultured with C-MSCs (NHLF (C-MSCs)) or with IPF (NHLF (IPF-MSCs)) for 72 h *: *p* < 0.05 NHLF (IPF-MSCs) vs. NHLF. (The * indicate significative differences referred to C-MSC (two-way ANOVA; *p* < 0.05).

Cell proliferation was evaluated after 72 h of culture. In mock samples, the proliferation of IPF-MSCs was significantly slower than C-MSCs (Figure 4A). After co-culture between C-MSCs and IPF-MSCs, cell counts were performed on both populations indicating that there were no significant differences in proliferation of co-cultured cells compared to mock cells (Figure 4A). Proliferation analysis of co-cultures of MSCs with NHLF revealed that IPF-MSCs slow down the proliferation of NHLF unlike C-MSCs (Figure 4B).

### 2.4. C-MSCs vs. IPF-MSCs: Expression of Genes Related to Inflammation

**Figure 5:**
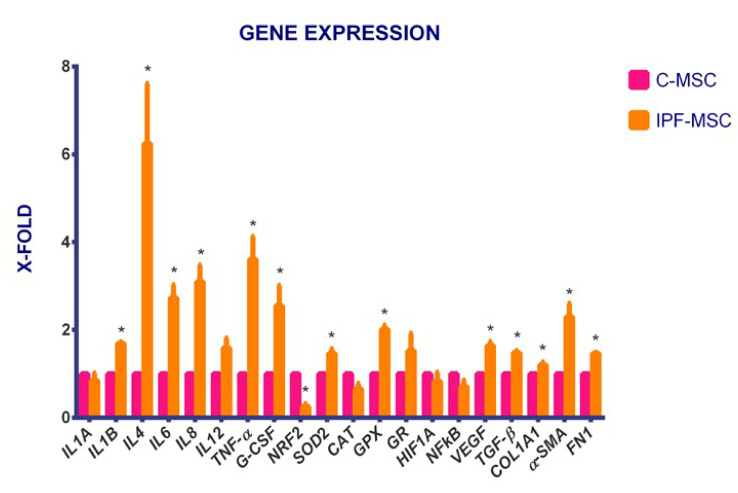
C-MSCs vs. IPF-MSCs: gene expression. The histogram displays the expression of selected genes referred specifically to inflammation (*IL1A*, *IL1B*, *IL4*, *IL6*, *IL8*, *IL12*, *TNF-α,* and *G-CSF*) and to other related pathways such as hypoxia (*HIF1A*, *NFkB,* and *VEGF*), oxidative stress (*SOD2*, *CAT*, *GPX*, *GR,* and *NRF2*) and ECM pathway (*TGF-β1*, *COL1A1*, *α-SMA,* and *FN1*) in C-MSCs and IPF-MSCs. Levels of expression detected in IPF-MSC are referred to as X-fold with respect to C-MSC (express as 1). Data are mean ± SD, over three independent experiments. The * indicates significative differences referred to C-MSC (one-way ANOVA; *p* < 0.05). C-MSCs: mesenchymal stem cells (MSCs) from controls; IPF-MSCs: MSCs from patients with IPF (idiopathic pulmonary fibrosis).

The expression of genes directly related to inflammation (*IL1A*, *IL1B*, *IL4*, *IL6*, *IL8*, *IL12*, *TNF-α* and *G-CSF*) and to other connected pathways such as hypoxia (*HIF1A*, *NFkB* and *VEGF*), oxidative stress (*SOD2*, *CAT*, *GPX*, *GR* and *NRF2*) and ECM pathway (*TGF-β1*, *COL1A1*, *α-SMA* and *FN1*) was evaluated by qRT-PCR. First, the analysis was referred only to C- and IPF-MSCs to assess potential differences caused by the pathological microenvironment. The results showed a general increase of the expression of all the selected genes in IPF-MSCs with respect to C-MSCs except for *NRF2* which is significantly downregulated (Figure 5). In detail, the expression of, *IL1B*, *IL4*, *IL6*, *IL8*, *TNF-α*, *G-CSF*, *SOD2*, *GPX*, *VEGF*, *TGF-β*, *COL1A1*, *α-SMA*, and *FN1* was significantly higher in IPF-MSCs than in C-MSCs.

### 2.5. Expression of Genes Related to Inflammation after Co-Cultures

**Figure 6:**
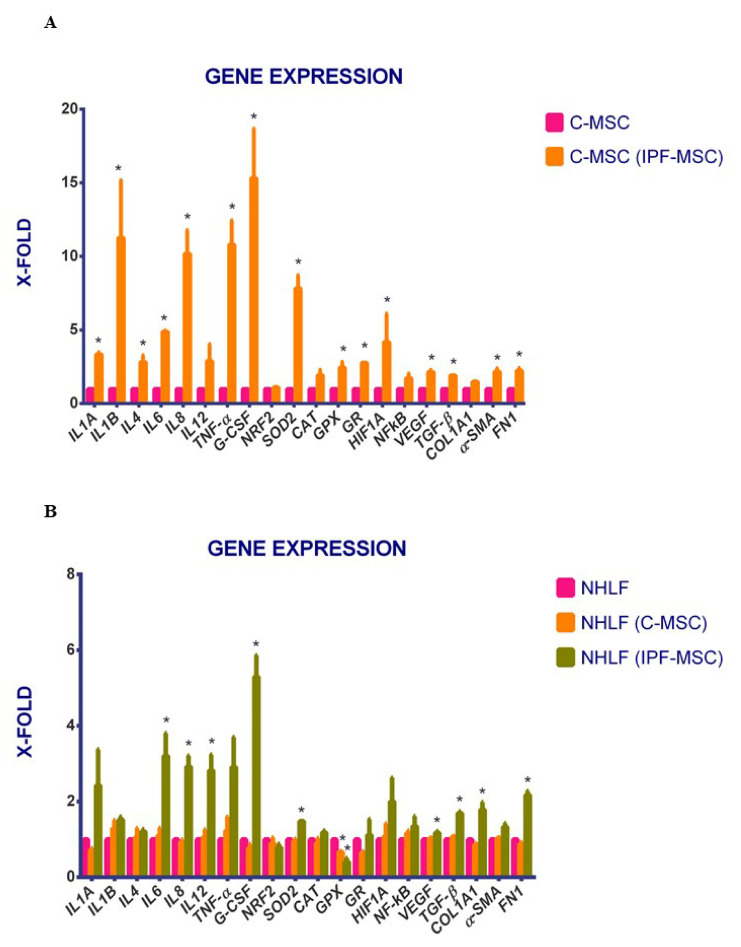
Gene expression after co-cultures. The histogram displays the expression of selected genes referred specifically to inflammation (*IL1A*, *IL1B*, *IL4*, *IL6*, *IL8*, *IL12*, *TNF-α,* and *G-CSF*) and to other related pathways such as hypoxia (*HIF1A*, *NFkB,* and *VEGF*), oxidative stress (*SOD2*, *CAT*, *GPX*, *GR,* and *NRF2*) and ECM pathway (*TGF-β1*, *COL1A1*, *α-SMA,* and *FN1*) after co-culture between C- and IPF-MSC (**A**). Levels of expression detected in C-MSC(IPF-MSC) is referred to as X-fold with respect to C-MSC (express as 1). The figure (**B**) shows the expression of the selected genes on NHLF before and after co-culturing with C-and IPF-MSCs. Levels of expression detected in NHLF(C-MSC) and in NHLF(IPF-MSC) are referred to as X-fold with respect to NHLF (express as 1). Data are mean ± SD, over three independent experiments. The * indicate significative differences referred to as C-MSC (one-way ANOVA; *p* < 0.05). C-MSCs: mesenchymal stem cells (MSCs) from controls cultured alone; C-MSC (IPF-MSCs): C-MSCs after 72 h of co-culture with IPF; NHLF: NHLF cultured alone; NHLF (C-MSCs): NHLF co-cultured with C-MSCs for 72 h; NHLF (IPF-MSCs): NHLF co-cultured with IPF-MSCs for 72 h.

Subsequently, the expression of the selected genes was analyzed after co-cultures performed between C- and IPF-MSCs or with NHLFs. C-MSCs co-cultured with IPF-MSCs (C-MSC (IPF-MSC)) showed significantly higher levels of expression of all the analyzed genes, and exception of *IL12*, *NRF2*, *CAT*, and *NFkB* whose increase was not statistically significant (Figure 6A), than C-MSCs cultured alone. No significant differences were instead detected in IPF-MSCs after co-culture with C-MSCs. Similar results were found after co-cultures with NHLF: while C-MSCs did not produce any relevant changes of expression on NHLF, the co-culture with IPF-MSCs caused a general increase in the expression of genes related to hypoxia, oxidative stress, and inflammation (Figure 6B).

### 2.6. C-MSCs vs. IPF-MSCs: Cytokines Secretion

**Figure 7:**
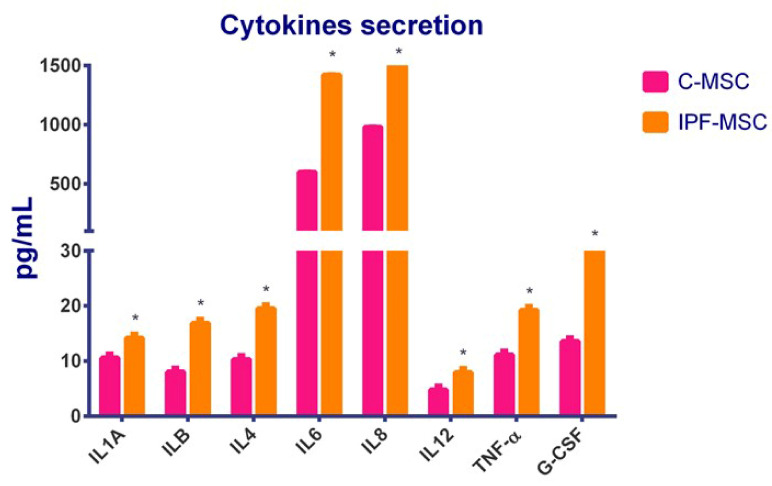
C-MSCs vs. IPF-MSCs: cytokines secretion. The secretion of IL1A, IL1B, IL4, IL6, IL8, IL12, TNF-α, and G-CSF was evaluated by ELISA. Levels of secretion were measured on collected medium after 72 h of culture and are referred to as pg/mL. Data are means ± SD from analyses performed on three separate experiments in triplicates. The * indicate significative differences between C-MSCs and IPF-MSC; (one-way ANOVA; *p* < 0.05). C-MSCs: mesenchymal stem cells (MSCs) from controls; IPF-MSCs: MSCs from patients with IPF (idiopathic pulmonary fibrosis).

An ELISA assay was performed to evaluate the secretion of cytokines directly related to inflammation (IL1A, IL1B, IL4, IL6, IL8, IL12, TNF-α, and G-CSF) in C- and IPF-MSCs. The results showed as the secretion of all the analyzed cytokines was significantly greater in IPF-MSC than in C-MSC (Figure 7) confirming data from qRT-PCR.

### 2.7. Cytokines Secretion after Co-Cultures

**Figure 8:**
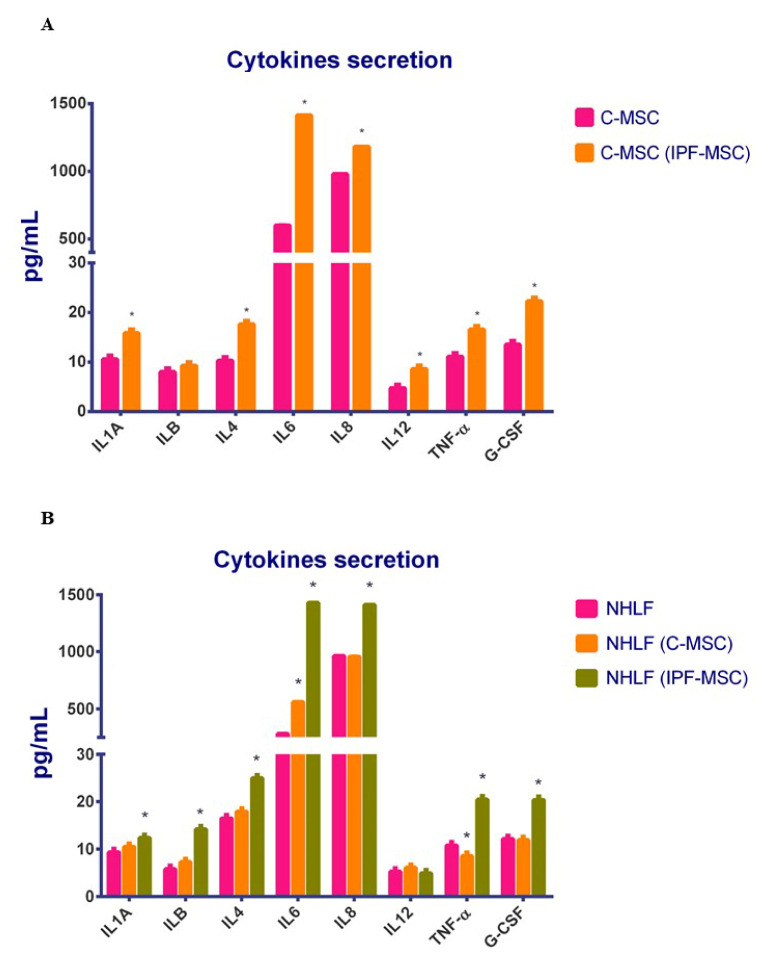
Cytokines secretion after co-cultures. The secretion of IL1A, IL1B, IL4, IL6, IL8, IL12, TNF-α, and G-CSF was evaluated by ELISA after co-culture between C- and IPF-MSC (**A**) and among NHLF and MSCs (**B**). Levels of secretion were measured on collected medium after 72 h of co-culture and are referred to as pg/mL. Data are means ± SD from analyses performed on three separate experiments in triplicates. In figure (**A**) the * indicates significative differences between C-MSCs and C-MSC(IPF-MSC); in figure (**B**) the * indicates significative differences between NHLF and NHLF(C-MSC) and between NHLF and NHLF(IPF.MSC); (one-way ANOVA; *p* < 0.05). C-MSCs: mesenchymal stem cells (MSCs) from controls cultured alone; C-MSC (IPF-MSCs): C-MSCs after 72 h of co-culture with IPF; NHLF: NHLF cultured alone; NHLF (C-MSCs): NHLF co-cultured with C-MSCs for 72 h; NHLF (IPF-MSCs): NHLF co-cultured with IPF-MSCs for 72 h.

After co-cultures between MSCs, as observed for the gene expression, IPF-MSCs induced a significant increase of secretion by C-MSCs of all the analyzed cytokines, except for IL1B (Figure 8A). Again, no significant changes were observed in IPF-MSCs after co-cultures with C-MSCs. The different ability to exert effects on cytokines secretion of IPF-MSCs compared to C-MSCs was confirmed in co-cultures with NHLF: only after co-culture with IPF-MSCs the level of the secreted cytokines was significantly higher than in mock NHLF (Figure 8B).

## 3. Discussion

Despite recent advances in the comprehension of IPF pathogenesis, the involvement of MSCs on IPF onset and development is still completely unknown. The aim of this study was to understand the effects of pathological microenvironment on MSCs and their consequent role in the onset of this pathology. First, cells isolated from control (C-MSCs) and fibrotic lung (IPF-MSCs) were characterized [20], confirming the undifferentiated nature. The tissue fragments used to develop human MSCs from IPF free controls were obtained from three different patients affected by early stage adenocarcinoma of the lung. Although a pathogenetic link between IPF and lung cancer has been supposed, due to the presence of mechanism such as EMT in both the diseases, these conditions are hugely different. Obtaining lung sample from patients with limited adenocarcinoma was considered the best option to gain tissue free from IPF, since sampling lungs of healthy subjects was not possible for ethical reasons, and tissue from patients with other more diffused lung diseases (secondary lung neoplasms, infections disease etc.,) was more likely to be impaired by the underlying extensive inflammation and damage.

The evaluation of stemness was integrated by the analysis of the expression of genes referred to as self-renew like SOX2, OCT4, NANOG, and KLF4. OCT4, SOX2 and NANOG form a reciprocal regulatory circuit whereby the equilibrium between stem cell self-renewal, proliferation, and differentiation is in perpetual balance [23]. No significant differences were found in the expression of these genes between the two groups. On the other hand, KLF4, known as another master player in self-renew and differentiation of stem cells, was downregulated in IPF-MSCs; this result is in line with those by Lin [24] that reported decreased level of KLF4 in lung tissues of human IPF and mouse models of bleomycin-induced pulmonary fibrosis. Several studies have shown that MSCs are affected by the surrounding microenvironment, becoming themselves protagonists of the disease development [25]; for this reason, MSCs were further characterized by IIF/ICC and qRT-PCR through the expression of α-SMA, collagen type 1 (COL1A1), fibronectin, and TGF-β1. Our analysis shows that their expression was stronger in IPF-MSCs than in C-MSCs and after co-cultures with IPF-MSCs, also C-MSCs and NHLF increased the levels of their expressions. These results are in contrast with those reported by Hostettler [17] that found only weak staining for α-SMA and fibronectin in MSCs isolated from fibrotic lung tissue. Particularly, TGF-β1 plays a central role in IPF, displaying different properties. First, TGF-β1 sustains fibroblast activation toward myofibroblasts and then it stimulates the expression of several pro-inflammatory and pro-fibrotic cytokines, such as TNF-α and IL6, thereby enhancing and perpetuating the fibrotic response [26]. The overexpression of α-SMA, collagen type 1, fibronectin, and TGF-β1 in IPF-MSCs confirms the influence of the microenvironment on lung fibrosis mesenchymal stem cells. IPF is a disease that occurs mainly in the elderly. This may suggest that cellular aging and senescence are responsible for the onset and progression of the disease [27]. Several studies report that IPF is characterized by increased senescence in both fibroblasts and alveolar epithelial cells [28,29] leading to cell cycle arrest [29]. In line with this, we found that IPF-MSCs had a significantly slower proliferation than C-MSCs. In addition, IPF-MSCs induce a lower proliferation of NHLFs after 72 h of co-culture. These results support the hypothesis that the senescent cells are themselves primarily characterized by an irreversible arrest of the cell cycle, but at the same time they remain metabolically active, leading to the development of a dynamic pro-inflammatory secretory profile that can affect the senescence and proliferation of neighboring cells [28,29,30]. To date, the long debate about the role of inflammation in the onset and development of IPF is still open. Even if immunosuppressive therapies are no longer indicated because of the negative results of the PANTHER trial [8,31], it is well accepted that IPF is characterized by the presence of a nontraditional inflammatory state maintained by cytokines, growth factors, and other mediators involving innate and adaptive immunity [32]. In the last few years, many studies tried to clarify the role of MSCs in inflammatory processes. It has been seen that MSCs can modulate the cells of the innate and adaptive immune system by secreting cytokines [33]. In this scenario, the expression and secretion of selected inflammatory cytokines such as IL1A, IL1B, IL4, IL6, IL8, IL12, TNF-α, and G-CSF were analyzed in C-MSCs, IPF-MSCs, and NHLF before and after co-cultures. Among mock samples, IPF-MSCs generally exhibited significantly higher expression and secretion of these cytokines than C-MSCs and NHLF, confirming that the inflamed microenvironment affects the behavior of MSCs. Co-cultures with IPF-MSCs were able to induce a strong increase in their expression\secretion both in C-MSC and NHLF, showing an ability to drive control cells toward a pathological phenotype. The over-production is particularly evident for IL6 and IL8 whose effects in IPF onset have been already identified [34,35]; G-CSF is another overexpressed cytokines and it is involved in the production and maturation of granulocytes [36], including macrophages that are the predominant cell type among the inflammatory ones in the lung after agent-induced IPF in mice [37]. To strengthen the hypothesis that the inflamed microenvironment may affect IPF-MSCs, other pathways interdependent with inflammation, such as hypoxia and oxidative stress (that are known as fibrotic inducers in IPF), were considered.

Oxidative stress can play a key role in the development of IPF by the increasing ROS and consequent imbalance in the production of antioxidant enzymes [38]. To evaluate if the microenvironment can induce an oxidative response, the expression of some enzymes with antioxidant activity such as SOD2, CAT, GPX, and GR was evaluated. Results showed a general overexpression of these genes in IPF-MSC compared to C-MSCs; notably, after co-culture with IPF-MSCs, C-MSCs express significantly higher level of genes than IPF-MSCs. The increase of the expression of these genes could therefore indicate an increase in intracellular stress with a relative overexpression of these enzymes in the attempt to counteract the damage.

NRF2 is known to protect against the development of pulmonary fibrosis by regulating the cellular redox level and lung Th1/Th2 balance [39]. In addition, it has been reported as MSC can operate on endoplasmic reticulum stress via the PERK-Nrf2 pathway in bleomycin-induced model [40]. Notably, its expression was lower in IPF-MSCs than in C-MSCs, confirming again as MSCs are influenced by the pathological microenvironment that acts to maintain itself. Nevertheless, co-culture with IPF-MSCs does not induce a decrease of this key molecule in the other cell types, suggesting that other mechanisms should be more functioning in perpetuating inflammation. Likewise, many recent publications implicate hypoxia-elicited inflammation, or inflammation during hypoxic conditions in the outcomes of a wide array of human diseases: inflammatory disease states are frequently characterized by tissue hypoxia but similarly, disease conditions that are primarily caused by a lack of oxygen are characterized by secondary inflammatory changes [10]. The expression of HIF1A gene was almost the same in C- and IPF-MSCs whereas VEGF was significantly more expressed by the IPF-MSCs. Our results are in contrast with other studies in which HIF1A expression was increased [41] but these were referred to as fibroblasts instead of undifferentiated cells.

Although some aspects are yet to be clarified, and further studies on animal model are necessary to evaluate the underlying mechanisms, in conclusion we can say that the pathological microenvironment affects the MSCs behavior: IPF-MSCs present a dysregulated expression/secretion of molecules related to ECM, inflammation, and interdependent pathways. In addition, when co-cultured with control cells, IPF-MSCs are able to drive an exceeding response by the other cell types moving them toward a pathological phenotype.

## 4. Material and Methods

### 4.1. Patients Enrollment

The study was conducted in collaboration with the Pulmonology and Thoracic Unit of Azienda Ospedaliero-Universitaria “Ospedali Riuniti” (Italy); all subjects gave their informed consent for inclusion before they participated in the study. The study was conducted in accordance with the Declaration of Helsinki, and the protocol was approved by the Institutional Ethics Committee of Università Politecnica delle Marche (2016-0360OR, 21-07-2016). Three patients with a final multidisciplinary diagnosis of IPF undergoing transbronchial lung cryobiopsy (TBLC) for diagnostic purpose according to current guidelines [42] were enrolled. TBLC was performed following the standardized international recommendation [43]. Three tissue samples from patients undergoing left lower lobectomy for early stage adenocarcinoma were used as IPF free controls. The final histopathological examination did not reveal any IPF pattern within the healthy lung tissue surrounding the adenocarcinoma nodules allowing to consider them healthy limited to the IPF pathology. The main demographical and clinical characteristics of both cases and controls are summarized in Table 2.

### 4.2. Isolation and Characterization of Mesenchymal Stem Cells from Healthy and Fibrotic Lung

Tissue fragments were cultured with MSCGM medium (Lonza, Basel, Switzerland) [43]. MSCs isolated from patients affected by idiopathic pulmonary fibrosis were named IPF-MSCs, while MSCs isolated from control lung of patients with adenocarcinoma were defined C-MSCs. According to the criteria by Dominici [20], cells were characterized by testing the plastic adherence, the immunophenotype and the multipotency as previously described [21,44]. Finally, the expression of genes related to stemness (*OCT4*, *SOX2*, *NANOG*, *KFL4*) was analyzed by qRT-PCR. Cell pellet was collected from 3 × 10^5^ cells after 72 h of culture and total RNA was extracted with Total RNA Purification Plus Kit (Norgen, Biotek Corp, Schmon Parkway, Thorold, ON, Canada). cDNA synthesis was performed using 5× All-In-One RT MasterMix (Applied Biological Materials, Richmond, BC, Canada). After amplification qRT-PCR was carried out with SsoFast EvaGreen Supermix (Bio-Rad, Milano, Italy) and fluorescence and melting curves were acquired. The parameter threshold cycle (Ct) was defined as the cycle number at which the first detectable increase above the threshold in fluorescence was observed. All samples were tested in duplicate with the housekeeping genes GAPDH for data normalization. mRNA expression was calculated by the 2^−ΔΔCt^ method [45]. The primer sequences are summarized in Table 3.

### 4.3. Production of α-SMA, Collagen Type 1, Fibronectin, and TGF-β1

For indirect immuno-fluorescence (IIF), 1.5 × 10^4^ cells were incubated with anti-collagen type I (COL1A1), anti-cellular fibronectin, and anti-α-SMA followed by goat anti-mouse FITC-conjugated antibody. Nuclei were visualized using Hoechst 33342 (all from Sigma-Aldrich, Milano, Italy). For immuno-cytochemistry (ICC), 1.5 × 10^4^ cells were incubated with anti-TGF-β1 (Sigma-Aldrich) and then immune-stained using the streptavidin–biotin–peroxidase technique (Dako Cytomation, Milano, Italy) and incubated with 3,3-diaminobenzidine. Slides were counterstained with Mayer’s hematoxylin. To quantify the expression of the proteins, the percentage of area occupied by the protein inside the cells was calculated using Fiji-ImageJ software (available online at https://imagej.net/Fiji) [22].

### 4.4. NHLF Culture

The cell line NHLF (ATCC, Manassas, Virginia, PCS-201-013, Normal, Human Lung Fibroblast) was cultured in DMEM (Dulbecco’s modified Eagle’s medium)/Hams F-12 50/50 Mix (Corning, NY, USA), 10% FBS (Corning), 1% penicillin/streptomycin (Euroclone, Milano, Italy) at 37 °C and 5% CO_2_.

### 4.5. MSCs Co-Cultures

Co-cultures were performed between C-MSC and IPF-MSC to evaluate the paracrine effects that could have one above the other. Total of 3 × 10^5^ IPF-MSCs were seeded at the lower surface and then 3 × 10^5^ of C-MSC were added at the upper surface of a polycarbonate transmembrane filter (pore size 0.4  μm; BD Falcon) for 72 h [44]. In addition, co-culture between C-MSC and NHLF and between IPF-MSC and NHLF were performed to evaluate the effects of control and fibrotic MSCs on lung fibroblast. Total of 3 × 10^5^ of NHLF were seeded at the lower of surface and then 3 × 10^5^ of C- or IPF-MSCs were individually added at the upper surface of a polycarbonate transmembrane filter. After 72 h mock and co-cultured cells were counted with Countess Automated Cell Counter (Invitrogen, Thermo Fisher Scientific, Oslo, Norway), collected and stored to −80 °C. The conditioned medium after co-cultures was collected, filtered with 0.2 µm filters and stored to −80 °C.

### 4.6. Expression of Genes Related to Inflammation, Oxidative Stress, Hypoxya, and ECM Pathway

Pellets collected from mock and co-cultured cells were used to analyze the expression of genes referred specifically to inflammation (*IL1A*, *IL1B*, *IL4*, *IL6*, *IL8*, *IL12*, *TNF-α,* and *G-CSF*) and to other related pathways such as oxidative stress (*SOD2*, *CAT*, *GPX*, *GR,* and *NRF2*), hypoxia (*HIF1A*, *NFkB,* and *VEGF*), and ECM (*TGF-β1*, *COL1A1*, *α-SMA,* and *FN1*). The primer sequences are summarized in Table 3. mRNA expression was calculated by the 2^−ΔΔCt^ method [45].

### 4.7. ELISA of Inflammation-Related Cytokines

The secretion of cytokines related to inflammation (IL1A, IL1B, IL4, IL6, IL8, IL12, TNF-α, G-CSF) were investigated by ELISA test with Multi-Analyte ELISArray kit (Qiagen, Milan, Italy) following manufactured instructions as previously described [46]. ELISA test was performed on conditioned medium derived from mock and co-cultured cells. Absorbance was read at 450 and 570 nm. The level of each cytokine was calculated as pg/mL.

### 4.8. Statistical Analysis

All experiments were performed in triplicate. For statistical analysis it was used GraphPad Prism 6 Software. All data are mean ± SD. For two-sample comparisons, significance was calculated by unpaired *t*-Student’s test. While, for three-experimental group comparisons significance was calculated by one-way or two-way ANOVA. The significance was set at *p* < 0.05.

## Figures and Tables

**Table 1:** Quantification of the protein expression.

	C-MSCs	IPF-MSCs
FIBRONECTIN %	44.59 ± 9.596	89.98 ± 4.539 *
COL1A1 %	12.47 ± 2.529	77.19 ± 4.440 *
α-SMA %	16.09 ± 7.094	76.58 ± 4.690 *
TGF-β %	4.757 ± 0.793	32.73 ± 5.046 *

Quantification of proteins expression processed by Fiji-ImageJ. Protein expression is represented by the percentage of the area it occupies inside the cell. The * indicates significative differences between C-MSCs and IPF-MSC (unpaired *t*-test; *p* < 0.05).

**Table 2:** Demographical and functional characteristics of IPF cases and controls.

Characteristics	IPF	CTRL
Age, years	70.3 ± 6.4	61.7 ± 0.5
Sex, no. male/no. female	1/2	0/3
Smoking status (no. Never/Ever)	1/2	2/1
Pulmonary function test at baseline, % predicted		
FEV1	85.0 ± 31.0	89.5 ± 14
FVC	83.3 ± 38.3	98.5 ± 19.5
DLco	56.0 ± 4.0	68 ± 17

IPF: patients affected by Idiopathic Pulmunary Fibrosis; CTRL: patients undergoing to lobectomy for adenocarcinoma; FEV1 = Forced expiratory volume in the 1st second; FVC: forced vital capacity; DLco: diffusing capacity of the lung for carbon monoxide.

**Table 3:** Primer sequences.

GENE	FORWARD 5′–3′	REVERSE 5′–3′
*GAPDH*	CCCTTCATTGACCTCAACTACATG	TGGGATTTCCATTGATGACAAGC
*SOX2*	ACACCAATCCCATCCACACT	GCAAACTTCCTGCAAAGCTC
*OCT4*	AGCGAACCAGTATCGAGAAC	TTACAGAACCACACTCGGAC
*NANOG*	TGAACCTCAGCTACAAACAG	CTGGATGTTCTGGGTCTGGT
*KLF4*	CCCACACAGGTGAGAAACCT	ATGTGTAAGGCGAGGTGGTC
*HIF1A*	GAAAGAGCCCGATGCCCT	TGATATGATCGTGTCCCCAGC
*NFKB*	AATGGTGGAGTCTGGGAAGG	TCTGACGTTTCCTCTGCACT
*VEGF*	CCTCCGAAACCATGAACTTT	ATGATTCTGCCCTCCTCCTTCT
*SOD2*	CTGGACAAACCTCAGCCCTAAC	AACCTGAGCCTTGGACACCAAC
*CAT*	GTGCGGAGATTCAACACTGCCA	TTCTCACACACGCGGCAATG
*GPX*	GTGCTCGGCTTCCCGTGCAAC	CTCGAAGAGCATGAAGTTGGGC
*GR*	TATGTGAGCCGCCTGAATGCCA	CACTGACCTCTATTGTGGGCTTG
*NRF2*	CAGCGACGGAAAGAGTATGA	TGGGCAACCTGGGAGTAG
*IL1A*	TCATTGGCGTTTGAGTCAGC	ACCACCATGCTCTCCTTGAA
*IL1B*	CGAATCTCCGACCACCACTA	AGCCTCGTTATCCCATGTGT
*IL4*	TTTGCTGCCTCCAAGAACAC	GTCGAGCCGTTTCAGGAATC
*IL6*	ATTCTGCGCAGCTTTAAGGA	AACAACAATCTGAGGTGCCC
*IL8*	GTGTGGGTCTGTTGTAGGGT	TCGGATATTCTCTTGGCCCT
*IL12*	AATGTTCCCATGCCTTCACC	CCAATGGTAAACAGGCCTCC
*TNF-α*	CGAGTCTGGGCAGGTCTACTTT	AAGCTGTAGGCCCCAGTGAGTT
*G-CSF*	GGACATGGTTTGACTCCCGA	CTTCCTTTCACACACAGGCC
*TGF-β1*	CAAGTGGACATCAACGGGTTC	TGCGGAAGTCAATGTAGC
*COL1A1*	GAGGGCCAAGACGACGAAGACATC	CAGATCACGTCATCGCACAAC
*α-SMA*	AGCCAAGCACTGTCAGGAATC	AGCCATTGTCACACACCAAGG
*FN1*	CGGGAGGCATTAGAAGGGAT	TTGCTTTGACTGACAGCCAC
